# Actinobacteria and Cyanobacteria Diversity in Terrestrial Antarctic Microenvironments Evaluated by Culture-Dependent and Independent Methods

**DOI:** 10.3389/fmicb.2019.01018

**Published:** 2019-05-31

**Authors:** Adriana Rego, Francisco Raio, Teresa P. Martins, Hugo Ribeiro, António G. G. Sousa, Joana Séneca, Mafalda S. Baptista, Charles K. Lee, S. Craig Cary, Vitor Ramos, Maria F. Carvalho, Pedro N. Leão, Catarina Magalhães

**Affiliations:** ^1^Interdisciplinary Centre of Marine and Environmental Research (CIIMAR/CIMAR), University of Porto, Porto, Portugal; ^2^Institute of Biomedical Sciences Abel Salazar (ICBAS), University of Porto, Porto, Portugal; ^3^International Centre for Terrestrial Antarctic Research, University of Waikato, Hamilton, New Zealand; ^4^School of Science, University of Waikato, Hamilton, New Zealand; ^5^Faculty of Sciences, University of Porto, Porto, Portugal

**Keywords:** actinobacteria, McMurdo Dry Valleys, Antarctic soil, bacteria diversity, bacterial cultivability, endolitic microbiota, Antarctic microenvironments, cyanobacteria

## Abstract

Bacterial diversity from McMurdo Dry Valleys in Antarctica, the coldest desert on earth, has become more easily assessed with the development of High Throughput Sequencing (HTS) techniques. However, some of the diversity remains inaccessible by the power of sequencing. In this study, we combine cultivation and HTS techniques to survey actinobacteria and cyanobacteria diversity along different soil and endolithic micro-environments of Victoria Valley in McMurdo Dry Valleys. Our results demonstrate that the Dry Valleys actinobacteria and cyanobacteria distribution is driven by environmental forces, in particular the effect of water availability and endolithic environments clearly conditioned the distribution of those communities. Data derived from HTS show that the percentage of cyanobacteria decreases from about 20% in the sample closest to the water source to negligible values on the last three samples of the transect with less water availability. Inversely, actinobacteria relative abundance increases from about 20% in wet soils to over 50% in the driest samples. Over 30% of the total HTS data set was composed of actinobacterial strains, mainly distributed by 5 families: *Sporichthyaceae*, *Euzebyaceae*, *Patulibacteraceae*, *Nocardioidaceae*, and *Rubrobacteraceae*. However, the 11 actinobacterial strains isolated in this study, belonged to *Micrococcaceae* and *Dermacoccaceae* families that were underrepresented in the HTS data set. A total of 10 cyanobacterial strains from the order Synechococcales were also isolated, distributed by 4 different genera (*Nodosilinea*, *Leptolyngbya*, *Pectolyngbya*, and *Acaryochloris*-like). In agreement with the cultivation results, *Leptolyngbya* was identified as dominant genus in the HTS data set. *Acaryochloris*-like cyanobacteria were found exclusively in the endolithic sample and represented 44% of the total 16S rRNA sequences, although despite our efforts we were not able to properly isolate any strain from this *Acaryochloris*-related group. The importance of combining cultivation and sequencing techniques is highlighted, as we have shown that culture-dependent methods employed in this study were able to retrieve actinobacteria and cyanobacteria taxa that were not detected in HTS data set, suggesting that the combination of both strategies can be usefull to recover both abundant and rare members of the communities.

## Introduction

The continent of Antarctica comprises about 0.34% ice-free areas (Convey, 2011) characterized by extreme cold and dry conditions (Wierzchos et al., 2004). In the McMurdo Dry Valleys (henceforth Dry Valleys), the largest ice-free region of the Antarctic continent (Pointing et al., 2009) and the coldest and driest desert on earth (Wood et al., 2008), the environmental stresses range from high variations in temperature (Doran et al., 2002), low nutrient availability and soil moisture (Buelow et al., 2016) to high ultraviolet solar radiation incidence (Perera et al., 2018).

Under such constraints, embracing the limits of physiological adaptability, microorganisms developed specialized strategies to survive, such as the colonization of edaphic and endolithic microenvironments (Walker and Pace, 2007), the entry into dormancy states (Goordial et al., 2017) and the biosynthesis of secondary metabolites (Wilson and Brimble, 2009; Tian et al., 2017).

Oligotrophic soils from the Dry Valleys are considered to be microbiologically distinct from all other soils worldwide (Fierer et al., 2012b) and High Throughput Sequencing (HTS) studies have proved their bacterial diversity is much larger than previously thought (Lee et al., 2012; Wei et al., 2016).

The Antarctica Dry Valleys soils are usually dominated by Actinobacteria, the prevalent phylum in cold arid soils (Pointing et al., 2009; Van Goethem et al., 2016; Goordial et al., 2017). Although the molecular basis behind actinobacteria dominance in cryoenvironments is still unknown (Goordial et al., 2015), metabolic activity at subzero temperatures has been detected (Soina et al., 2004). The formation of spores allows the survival in desert-like habitats (Mohammadipanah and Wink, 2016) and cyst-like resting forms have been described for non-sporulating actinobacteria species (Soina et al., 2004).

In addition to soil environments, rocky niches are of particular relevance in the Dry Valleys ecosystems since they provide protection to different biota from harsh environmental conditions such as intense solar radiation exposure, temperature fluctuations, wind, and desiccation (Cockell and Stokes, 2004; Walker and Pace, 2007). These rock-inhabiting organisms are very important in Dry Valleys because of the extent of rock-exposed surface, thus accounting largely for the productivity and biomass in this system (Omelon, 2008; Pointing and Belnap, 2012). Dry Valleys hypolithic and endolithic communities are often dominated by cyanobacteria (Omelon, 2008; Chan et al., 2012; Van Goethem et al., 2016). Indeed, as primary colonizers after the retreat of glaciers, cyanobacteria are leading components of Dry Valleys ecosystem, enabling colonization by other microorganisms (Vincent, 2000). They are well adapted to the stress of desiccation and despite most of the Dry Valleys soils lack any visible cyanobacterial growth, their presence was detected through HTS even in low moisture soil samples (Wood et al., 2008).

In fact, HTS techniques have revolutionized the traditional biodiversity studies based on culturing approaches and opened an array of new opportunities to explore previously hard to access environments, as are extreme environments. These approaches can be used to study both cultured and uncultured diversity, being of particular relevance in these types of environments – where the unique environmental conditions are hard to mimick in the laboratory. Cultivation-independent studies have revealed that in the Dry Valleys, abiotic factors drive the diversity and structure of microbial communities (Pointing et al., 2009; Lee et al., 2012; Magalhães et al., 2012). Aridity seem to be the most preponderant factor shaping bacterial communites, not only in Dry Valleys but across multiple deserts (Kastovská et al., 2005; Pointing et al., 2007; Wood et al., 2008). The type of habitat (lithic vs. soil) also has a preponderant role in shaping the bacterial community composition as shown for Antarctic and other hyper-arid deserts (Azúa-Bustos et al., 2011; Stomeo et al., 2013; Makhalanyane et al., 2015).

Although HTS techniques promised to be able to replace bacterial culturing (Venter et al., 2004), cultivation techniques are still necessary to improve taxonomic resolution and even diversity coverage (Lagier et al., 2015; Choi et al., 2017; Ramos et al., 2017), as well as to recover whole genomes or allow physiological and metabolic studies (Sørensen et al., 2002; Ramos et al., 2018; Lambrechts et al., 2019). The information provided by the bacterial isolates can further allow to understand the cultivation requirements and develop directed cultivation techniques leading to potential novel discoveries (Ramos et al., 2018; Tahon et al., 2018). Antarctic bacteria, by evolving in such extreme conditions are particularly interesting in terms of biotechnological applications, including for bioremediation (Gran-Scheuch et al., 2017; Lee et al., 2018), antimicrobials (Gesheva and Vasileva-Tonkova, 2012; Tedesco et al., 2016) and production of anti-freezing molecules (Muñoz et al., 2017).

Combined approaches encompassing culture dependent and independent techniques to retrieve a broader bacterial diversity have been employed for Antarctic studies (Babalola et al., 2009; Aislabie et al., 2013). However, only a fraction of the taxa recovered did overlap, highlighting the complementarity of both approaches. In a comprehensive study Lambrechts et al. (2019) have shown that over 85% of Antarctic soil bacterial sequences available in databases still belong to uncultured genera or higher taxonomic level. Even for extensively studied phyla, such as actinobacteria, there are reports of uncultured phylotypes (Smith et al., 2006; Babalola et al., 2009).

By mimicking natural conditions, novel micro-culturing techniques, including soil substrate membrane system (SSMS) (Pudasaini et al., 2017) and extended incubation times (Tahon and Willems, 2017) have reduced the gap between the cultured and uncultured approaches. Several Antarctic studies have successfully retrieved novel genera and families, including first isolates of novel taxa of recalcitrant bacteria, by employing novel cultivation approaches (van Dorst et al., 2016; Pulschen et al., 2017; Tahon and Willems, 2017; Tahon et al., 2018). In the present study, we combine HTS and microbial cultivation techniques as culture-independent and dependent approaches to survey actinobacteria and cyanobacteria diversity along different soil and endolithic micro-environments of Victoria Valley, one of the Dry Valleys. Novel cultivation techniques, previously fruitful in retrieving novel and recalcitrant taxa from Antarctic and other deserts, were included. A comprehensive insight of the main factors shaping endolithic and edaphic microbial communities, such as moisture levels and pH is addressed.

## Materials and Methods

### Samples Location and Collection

**FIGURE 1:**
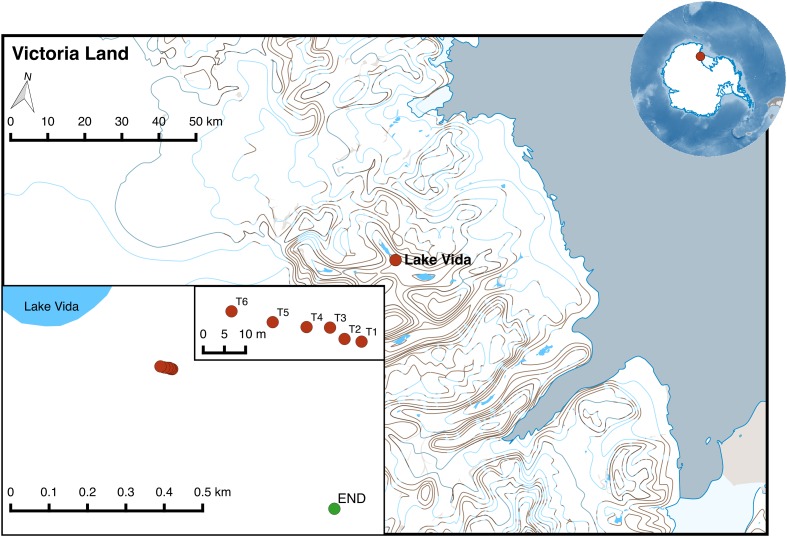
Location of sampling points in Victoria Valley (transect soil samples marked in red and endolithic sample in green). The map was generated using QGIS v2.8.2 and the Quantarctica data set (Matsuoka et al., 2018).

Substrate from a rock with endolithic colonization (END) and from a soil transect with a gradient of water availability were collected in Victoria Valley during the K020 Mission in January 2013, integrated in the NZTABS international program. For the transect, a total of six sites between T1 and T6 were sampled from a 32 m transect with increasing distance from a water pond near the main water source in the Victoria Valley – Lake Vida (Figure 1 and Table 1). Several scoops of soil were collected aseptically and stored in a sterile Whirl-Pak. All samples were kept at −30°C in Lifeguard solution (MoBio) in the 1st week after sample and then at −80°C until further analysis.

**Table 1:** Variation of soil characteristics across the Dry Valley soil transect, including water availability (Aw), pH and conductivity (cond).

Sample code	Latitude	Longitude	Aw	pH	Cond (μS/cm)
T1	S77 20.241	E161 38.593	1,06	7,09	61
T2	S77 20.240	E161 38.584	1,02	8,02	314
T3	S77 20.238	E161 38.578	1,01	7,36	2530
T4	S77 20.237	E161 38.565	0,6	8,39	21
T5	S77 20.235	E161 38.547	0,15	8,41	122
T6	S77 20.232	E161 38.526	0,28	8,39	21

### Physicochemical Analysis of Transect Soil Samples

Water Activity (Aw) was measured *in situ* in all sampling points using a portable water activity analyzer (PaWKit AquaLab, Decagon). Conductivity and pH were also measured in all soil samples using a CyberScan PC 510 Bench Meter (Eutech Instruments) following the slurry technique which consists in mixing 1:2.5 mass ratio of samples and de-ionized water (Edmeades et al., 1985).

### Bacterial Community Diversity Analysis

Environmental DNA (eDNA) was extracted using a modification of the CTAB extraction protocol (Barrett et al., 2006). The 16S rRNA gene was amplified by PCR using the universal primer pair 27F/1492R (Weisburg et al., 1991) and then sequenced by pyrosequencing technology. Briefly, the 16S rRNA gene was amplified for the V3-V4 hypervariable region with barcoded fusion primers containing the Roche-454 A and B Titanium sequencing adapters, an eight-base barcode sequence, the forward (5′–ACTCCTACGGGAGGCAG-3′) and reverse (5′–TACNVRRGTHTCTAATYC -3′) primers (Wang and Qian, 2009). The PCR reaction was performed using 5 U of Advantage Taq polymerase (Clontech), 0.2 μM of each primer, 0.2 mM dNTPs, 6% DMSO and 2–3 μL of template DNA. The PCR conditions employed were: initial denaturation step at 94°C for 3 min, followed by 25 cycles of 94°C for 30 s, 44°C for 45 s and 68°C for 60 s and a final elongation step at 68°C for 10 min. The amplicons were quantified by fluorimetry with PicoGreen (Invitrogen), pooled at equimolar concentrations and sequenced in the A direction with GS 454 FLX Titanium chemistry, according to the manufacturer’s instructions (Roche, 454 Life Sciences) at Biocant (Cantanhede, Portugal). The 454-machine-generated FASTA (.fna) and quality score (.qual) files were processed using the QIIME (Quantitative insights into microbial ecology) pipeline (Caporaso et al., 2010). Initially, raw reads were demultiplexed and subjected to a quality filtering – sequences with a quality score below 25 were removed. The next step, Pick OTUs (Operational Taxonomic Units) (Seath et al., 1973) was performed in parallel with 3 different workflows: pick_de_novos_otus.py, pick_otus.py (closed-reference method) and pick_open_reference_otus.py (open-reference method). The OTU table obtained from the open-reference method was selected for the downstream analyses. Essentially, all sequences were clustered into OTUs at 97% sequence similarity using UCLUST (Edgar, 2010) and the reads aligned to the Greengenes v13_8 (GG) (DeSantis et al., 2006) database using PyNAST. For the taxonomic assignment, the RDP Classifier 2.2 (Wang et al., 2007) was used with the UCLUST method. For each sample, alpha and beta diversity metrics were calculated using weighted and unweighted UniFrac metrics (Lozupone and Knight, 2005). The R packages phyloseq (Mcmurdie and Holmes, 2013) and ggplot2 (Wickham, 2009) were used for downstream analysis and visualization including alpha diversity calculations and relative and total abundance taxonomy summary charts.

### Isolation of Actinobacteria and Cyanobacteria

Some of the environmental samples that were collected in Victoria Valley were also used for bacterial isolation, based on their composition as estimated by HTS. Samples T5 and T6 from soil transect were selected for the isolation of actinobacteria, while endolithic and transect samples T1 and T3 were used for the isolation of cyanobacteria. In addition, sample T6 was also used for attempting to isolate low-abundance cyanobacteria in an actinobacteria-dominated sample. It is known that the cultivable fraction of the microbial richness is typically below 1% (Epstein, 2013), so in order to improve the cultivability and maximize the recovery of microbial strains from the samples, different culture strategies – including pre-treatments – were employed as described below.

### Culture Strategies for the Isolation of Actinobacteria

For the transect sample T6, 0.5 g of the original sample (soil) were weighted under sterile conditions and 5 mL of sterile saline solution (0.85% NaCl at the temperature of 4°C) were added to resuspend the sample. The solution was vortexed for 10 min and allowed to settle for 2 min in an ice bath. Sequential dilutions (down to a dilution factor of 10^−2^) of the supernatant were performed, inoculated (in duplicate) on solid media (as described below) and incubated at three different temperatures (4, 9 and 19°C). All the media were supplemented with 50 mg/L of cycloheximide (BioChemica) and streptomycin (BioChemica) to inhibit the growth of fungi or other eukaryotes and Gram-negative bacteria, respectively. The dilutions were plated onto an oligotrophic medium – Nutrient-poor sediment extract (NPS) – primarily made with an extract from the original Antarctica soil sample and then with sand collected from a beach in northern Portugal (Francelos Beach, Vila Nova de Gaia, Portugal), to simulate the oligotrophic environmental conditions. Previous works have indicated that soil-extract agar is able to retrieve a wider and more diverse range of biodiversity when compared to traditional media (Hamaki et al., 2005). Briefly, ca. 500 g of substrate was mixed with 500 mL of distilled water, homogenized and allowed to settle. For medium preparation, 100 mL of the supernatant solution was mixed with 900 mL of distilled water and 17 g of bacteriological agar. Obtained colonies were then streaked in the same medium and in richer media, in order to investigate which one could render a higher biomass growth. The richer media used were: modified nutrient-poor sediment extract (MNPS): 5 g/L soluble starch, 1 g/L potassium nitrate, 100 mL/L substrate extract and 17 g/L agar; International Streptomyces Project medium 2 (ISP2) (Shirling and Gottlieb, 1966) and raffinose histidine agar (RH) (Vickers et al., 1984). Bacterial colonies were successively streaked until pure colonies were achieved.

For the transect sample T5, a more selective approach was used. The soil sample (0.5 g) was weighted under sterile conditions and 2.5 mL of sterile saline solution were added to resuspend the sample. The solution was incubated on an ultrasound for 1 min and vortexed for 5 min. The sample was then submitted to two different pre-treatments to maximize the selection of sporulating actinobacteria: (1) heat-shock, which consisted in the incubation of 1 mL of the suspension at 50°C, for 5 min and (2) incubation with antibiotics (Hameş-Kocabaş and Uzel, 2012), consisting in the incubation of 1 mL of the suspension with 20 mg/L of streptomycin (BioChemica) and nalidixic acid (BioChemica), at 28°C for 30 min. For each pre-treatment, serial dilutions (down to a dilution factor of 10^−2^) were performed and plated onto different media selective for actinobacteria: Actinomycete Isolation Agar (AIA): sodium caseinate 2 g/L, L-asparagine 0.1 g/L, sodium propionate 4 g/L, dipotassium phosphate; Czapeck agar (Axenov-Gribanov et al., 2016) and Starch Casein Nitrate Agar (SCN): 10 g/L soluble starch, 0.3 g/L casein sodium salt from bovine milk, 2.62 g/L potassium phosphate dibasic trihydrate, 2 g/L potassium nitrate, 2 g/L sodium chloride, 0.05 g/L magnesium sulfate heptahydrate, 0.02 g/L calcium carbonate, 0.01 g/L iron(II)sulfate heptahydrate. The plates were incubated at 4 and 28°C. The bacterial colonies grown in the plates were streaked in the same isolation media until pure colonies were obtained.

### Culture Strategies for the Isolation of Cyanobacteria

The endolithic (END) and samples T1, T3, and T6 of the soil transect, all preserved at −80°C in Lifeguard solution, were used for cyanobacterial isolation. Before the inoculation, samples were submitted to a washing process, which consisted in the centrifugation of the samples at 4500 × *g* for 3 min, removal of the supernatant, resuspension of the pellet in BG11_0_ medium [without nitrogen source (Rippka, 1988)], brief agitation of the suspension, centrifugation again of the suspension and discarding of the supernatant. The inoculation was carried out by adding an equal part of the pelleted samples to (a) glass Erlenmeyer’s of 100 mL with liquid medium [BG11_0_ and Z8 (Kotai, 1972)] and (b) to solid (Z8 and BG11_0_) agar plates, and allowed to grow at 19°C, under a 12:12 h light (20–30 μmol m^−2^ s^−1^ photon irradiance):dark cycle. Plates were prepared with 1.5% agarose and supplemented with 0.5% of cycloheximide (5 mL/L), to prevent the growth of eukaryotic microorganisms. When visible growth was detected in the liquid/solid media, aliquots were transferred and streaked onto solid Z8 or BG11_0_ medium plates. The single colonies were selected and re-streaked aseptically to fresh Z8 and/or BG11_0_ medium plates. The procedure was repeated until isolation was achieved (Rippka, 1988). cyanobacterial isolates were visually inspected under a microscope (Leica DMLB) and then transferred to Z8, both in liquid and solid (agar) medium. Isolates are maintained in the LEGE Culture Collection (Ramos et al., 2018).

### Identification of Bacterial Isolates Through 16S rRNA Gene Sequence Amplification

After isolation of pure cultures, each bacterial isolate was grown in 10 mL of liquid medium (composition according to the medium from which the isolated was retrieved) in 50 mL Falcon tubes/100 mL Erlenmeyer’s, until enough biomass was obtained to extract DNA. DNA was extracted using the E.Z.N.A.^®^Bacterial DNA Kit (OMEGA bio-tek) and Purelink Genomic DNA Mini Kit – Gram-negative bacterial cell protocol (Invitrogen), for bacterial and cyanobacterial strains, respectively. The manufacturer’s instructions were followed, and DNA was eluted in 100 μL of elution buffer. The integrity of the gDNA was assessed by agarose gel electrophoresis (0.8% agarose gel prepared in TAE buffer 1X, stained with 1 μL of SYBR^®^Safe DNA Gel Stain from Thermo Fisher Scientific). One microliter of DNA (with loading dye) was loaded onto each lane before electrophoresis at 80 V for 30 min. The 16S rRNA gene was amplified by PCR in a Veriti^®^96-Well Thermal Cycler (Thermo Fisher Scientific) using primer pair 27F/1492R (Weisburg et al., 1991) (1465 bp) for bacteria and primer pairs CYA106F, CYA359F, CYA781R (Nubel et al., 1997) and 1492R (Weisburg et al., 1991) (combinations CYA106F – CYA781R: ∼675 bp and CYA359F – 1492R: ∼1130 bp) for cyanobacteria.

For bacteria, the PCR reaction was prepared in a volume of 10 μL containing 1× TaKaRA PCR Buffer (TAKARA BIO INC.), 1.5 mM MgCl_2_ (TAKARA BIO INC.), 250 μM dNTPs (TAKARA BIO INC.), 1.5 μL of each primer (2 μM), 0.25 mg/mL of UltraPure^TM^ BSA (Life Technologies), 0.25 U TaKaRa Taq^TM^ Hot Start Version (TAKARA BIO INC.) and 1 μL of template DNA. The PCR conditions were: initial denaturation step at 98°C for 2 min, followed by 30 cycles of a denaturation step at 94°C for 30 s, annealing at 48°C for 90 s and extension at 72°C for 2 min, followed by a final extension step at 72°C for 10 min. PCR products (3 μL loaded in each well) were separated by electrophoresis on a 1.5% (w/v) agarose gel during 30 min at 150 V. The ladder utilized was GRS ladder 1 kb (Grisp). The gel was stained with 1 μL SYBR^®^Safe DNA Gel Stain (Thermo Fisher Scientific), visualized under UV-light at Gel Doc XR+ System (BIO-RAD) and analyzed with the Image Lab^TM^ software (BIO-RAD). For cyanobacteria, the PCR reaction was prepared in a volume of 20 μL containing 1× Green GoTaq^®^Flexi Buffer (Promega), 2.5 mM MgCl2 (Promega), 500 μM of DNTP Mix (Promega), 0.1 μM of each of the primers, 0.5 U of GoTaq^®^DNA Polymerase (Promega) and 2 μL of template DNA. The PCR conditions executed were: initial denaturation step at 92°C for 4 min, followed by 35 cycles of a denaturation step at 92°C for 30 s, annealing at 50°C for 30 s and extension at 72°C for 1 min, followed by a final extension step at 72°C for 5 min.

The PCR products of bacteria and cyanobacteria were then sequenced by Sanger sequencing at i3S (Porto, Portugal) and GATC Biotech (Constance, Germany), respectively. Raw forward and reverse sequences (ab1 files) were imported into Geneious 8.1.9 (Kearse et al., 2012) for *de novo* assembling.

### Phylogenetic Analysis of Bacterial Isolates

The obtained sequences (approximately 1,400 and 1,100 bp for actinobacteria and cyanobacteria, respectively) were submitted to a blast(n) analysis against the NCBI Nucleotide collection database and the sequences from the first 5 blast(n) matches were retrieved. The multiple sequence alignment (using the ClustalW algorithm) and the phylogenetic analysis were performed in MEGA7 (Kumar et al., 2016). The alignments were manually curated to remove short sequences and gap regions. The best nucleotide substitution model was determined by the corrected Akaike Information Criterion (AICc) in MEGA7. The phylogenetic trees were reconstructed using the Maximum Likelihood statistical method, bootstrap (with 500 replications) and the correspondent best nucleotide substitution model (TN93 + G and GTR + G + I).

## Results and Discussion

The hyper-arid desert of McMurdo Dry Valleys, located in Victoria Land is considered one of the most inhospitable habitats, being restricted to microbial colonization (Pointing et al., 2009). In these environments, abiotic factors such as moisture, pH and conductivity, clearly drive the diversity and structure of the microbial communities (Pointing et al., 2009; Magalhães et al., 2012; Magalhães et al., 2014).

In this study, a transect of soil samples was collected in Victoria Valley, with a clear decrease in water availability (AW) from the wetter sampling sites (T1, T2, and T3) to the drier ones (T5 and T6) (Table 1). Soil pH ranged from neutral (7.09) to moderately alkaline (8.41), increasing with the distance to water availability.

### Cyanobacteria and Actinobacteria Diversity and Distribution

The bacterial community composition across the transect was assessed through 454 pyrosequencing of the 16S rRNA gene. A total of 180499 sequences were obtained for the seven studied samples, which after quality filtering decreased to 71447. The number of sequences per sample ranged between 2950 (endolithic sample) to 17570 (sample T6). In total 4530 different OTUs (at 97% identity) were retrieved (Supplementary Table S1).

**FIGURE 2:**
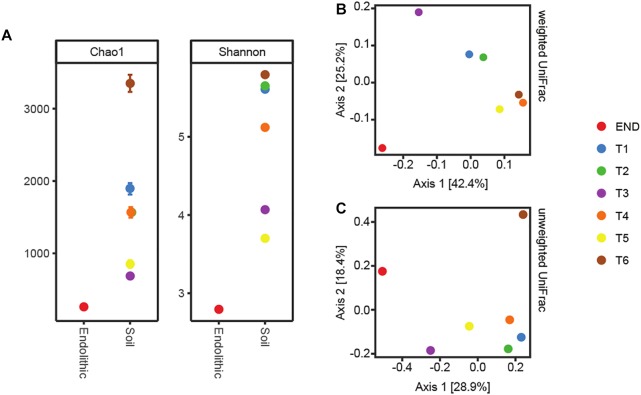
**(A)** Alpha-diversity metrics Chao1 and Shannon. **(B)** PcoA plots using the weighted UniFrac metrics. **(C)** PcoA plots using the unweighted.

Alpha-diversity results indicated that bacterial diversity was not fully covered from the sequencing effort in transect samples as a plateau phase was not reached, with the exception of the endolithic sample (Supplementary Figure S1). Two different beta-diversity metrics were employed – weighted and unweighted UniFrac (Lozupone and Knight, 2005), both phylogeny-based. The resultant output was summarized by Principal Coordinates Analysis (PCoA) (Figure 2B,C). The principal coordinate 1 (PC1) explained 28.9% and 42.4% of the amount of variation for unweighted and weighted analyses, respectively. From both plots, it is observable a similar clustering pattern – samples T1 and T2 cluster together, as well as samples T4, T5, and T6. Further, sample T3 and END seem to be distributed separately from the other samples. This clustering pattern may be indicative of a switch in habitat type from moisture soils, comprising T1 and T2, which were the ones closer to the water source, to open arid soils, comprising locations T4, T5, and T6 and correspond to the locations which are further away from the water source.

**FIGURE 3:**
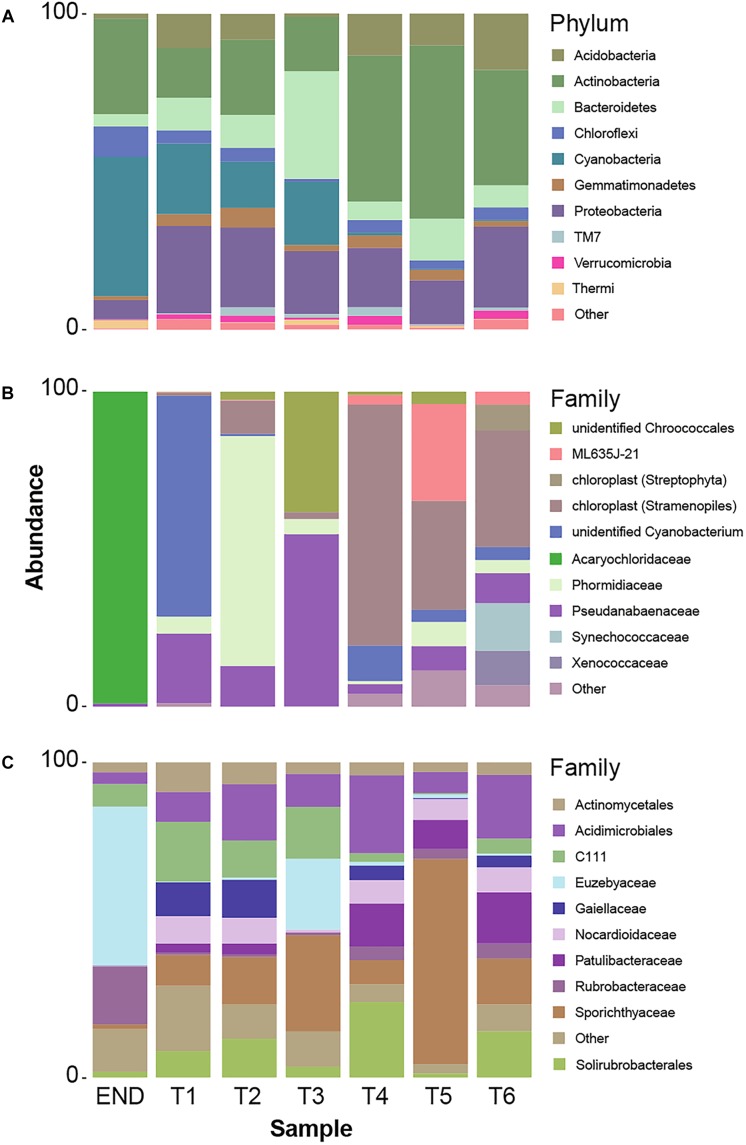
**(A)** Taxonomy summary bar chart of relative frequency of 10 most abundant phyla per sampling point. **(B)** Summary bar chart of 10 most abundant taxonomic frequency distributions at Family level from cyanobacteria phylum. **(C)** Summary bar chart of 10 most abundant taxonomic frequency distributions at Family level from actinobacteria phylum.

In accordance with previous reports for Antarctic (Pointing et al., 2009; Van Goethem et al., 2016) and hot hyperarid deserts (Azúa-Bustos et al., 2011; Stomeo et al., 2013; Makhalanyane et al., 2015), the type of habitat (endolithic vs. soil) dramatically constrains bacterial community composition. Clearly distinct taxonomic and phylogenetic composition was observed in the two niches under study (Figure 3). Also consistent with previous studies (Van Goethem et al., 2016), diversity indices (alpha-diversity) revealed soil samples as more diverse than the endolithic sample, according to the number of observed OTUs (Supplementary Figure S1) and the richness and diversity of the sample (Figure 2A). A total of 34 bacterial phyla were detected across all the samples, with the phyla Actinobacteria, Proteobacteria, Cyanobacteria, and Bacteroidetes being the most abundant and present in all samples (Figure 3A). Actinobacteria, Acidobacteria, and Bacteroidetes are usually the dominant phyla described for Dry Valleys (Aislabie et al., 2006; Cary et al., 2010). Interestingly, in contrast to previous studies (Smith et al., 2006; Fierer et al., 2012b; Van Goethem et al., 2016), Proteobacteria was highly represented on the transect and endolithic samples. This phyla is generally dependent on high organic soil contents, which is not the case of most oligotrophic Antarctic soils (Cary et al., 2010; Chan et al., 2013). As expected, Acidobacteria, considered an oligotrophic phylum (Fierer et al., 2012b), was also well represented and dispersed among the different samples (Figure 3A).

In this study, special attention was given to the distribution of Actinobacteria and Cyanobacteria – the most abundant heterotrophic and autotrophic phylum, respectively.

Cyanobacteria are usually the dominant phyla in lithic-associated communities (Chan et al., 2012) across multiple deserts (Pointing et al., 2007; Crits-Christoph et al., 2016; Khomutovska et al., 2017; Perera et al., 2018). In this study cyanobacteria close related to *Acaryochloris* were found exclusively in the endolithic sample and represented 44% of the total relative abundance (Figure 3B). Up till now, the two described species of *Acaryochloris* are marine (Miyashita et al., 2003; Partensky et al., 2018) but cyanobacteria closely related to these taxa have already been associated with endolithic communities in Antarctic Dry Valleys and Atacama granite and calcite rocks, respectively (de Los Ríos et al., 2007; Crits-Christoph et al., 2016), and within the underexplored cold desert of Pamir mountains (Khomutovska et al., 2017). These niches provide a barrier to penetration of organisms, protection against harmful solar irradiance as well as a microclimate distinct from the exterior of the rock, with higher moisture levels (Friedmann, 1980; de Los Ríos et al., 2007; Wierzchos et al., 2012). On the other hand, one of the main factors that influence the composition of endolithic photosynthetic communities is the quantity and quality of light available (de Los Ríos et al., 2005). Curiously, the same applies for the distribution of *Acaryochloris* in marine environments (Chan et al., 2007; Partensky et al., 2018). This genus is characterized by having sheathed and non-motile cells, but its chief distinctive character is the presence of chlorophyll *d* (chl *d*) as the major photosynthetic pigment (Miyashita et al., 2003; Partensky et al., 2018).

**FIGURE 4:**
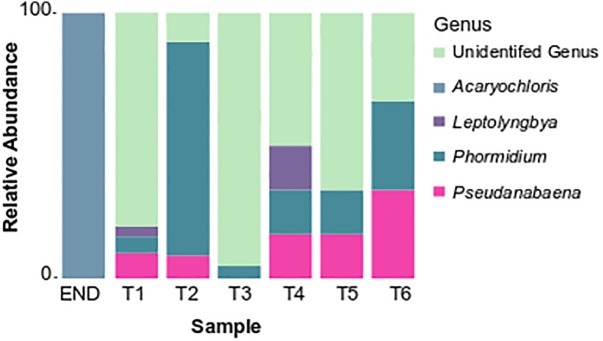
Taxonomy summary bar chart of relative frequency of the most abundant cyanobacterial genus.

Over 30% of the total bacterial relative abundance in the current dataset was attributable to actinobacterial strains, mainly distributed by two families – *Euzebyaceae* and *Rubrobacteraceae* (Figure 3A,B). Members of *Euzebya* genus have been reported from Victoria Valley’s hypolithic and endolithic communities (Van Goethem et al., 2016). The *Rubrobacter* genus, commonly found in Dry Valleys (Wei et al., 2016) has been reported as dominant genus of endolithic microbial communities in Atacama Desert (Crits-Christoph et al., 2016; Meslier et al., 2018). Water availability seemed to clearly define a threshold or a limit for colonization of some bacterial phyla, mainly cyanobacteria, along the soil transect. The HTS data show that the percentage of cyanobacteria decreases from about 20% in sample T1 to negligible values on the last three samples of the transect (T4–T6) (Figure 3A). On sample T1, *Leptolyngbya* and *Pseudanabaena* genera were well represented while in sample T2, the genus *Phormidium* was the most abundant (Figure 4). *Leptolyngbya* is usually associated with lake and maritime Antarctic communities (Taton et al., 2006; Zakhia et al., 2008), while *Phormidium* is commonly found in Antarctic water-saturated soils and river beds (Vincent et al., 1993) but both were found in polar deserts (Michaud et al., 2012). These findings suggest, as already shown for Antarctic (Pointing et al., 2007; Wood et al., 2008; Van Goethem et al., 2016) and other hyper-arid deserts (Kastovská et al., 2005), that water availability and thus distance to aquatic ecosystems shapes the taxonomic composition of the cyanobacterial communities. In fact, in hyper-arid habitats, water availability was shown to be the main driver and most limiting environmental factor for cyanobacteria distribution (Warren-Rhodes et al., 2006, 2007). In agreement, the results obtained in this study suggest the existence of a biological threshold for cyanobacterial colonization and survival, observed between 1–0.6 of water availability (Aw), while moving from sample T3–T4 (Table 1). Limited photosynthetic activity is related to limiting moisture levels (Tracy et al., 2010) and might be a reason for cyanobacterial activity reduction and distribution.

Among cyanobacteria, Synechococcales (represented by the genera *Acaryochloris*, *Leptolyngbya*, and *Pseudanabaena*) and Oscillatoriales (*Phormidium*) orders dominated on the studied samples. Interestingly, *Chroococcidiopsis* (Caiola et al., 1993) a desiccation tolerant cyanobacteria, dominant in arid and hyper-arid deserts (Cámara et al., 2015; Khomutovska et al., 2017; Lacap-Bugler et al., 2017; Gómez-Silva, 2018) was not detected in this study.

Previous studies have stated that Oscillatoriales are capable of overcoming Chroococcidiopsales in certain cold desert conditions, namely *Phormidium* have been suggested to pursue a competitive advantage in colonizing cold-habitats (de la Torre et al., 2003; Pointing et al., 2007). This group is also commonly associated with places with higher availability of water, as described by Steven et al. (2013) in Arctic desert soils. Filamentous cyanobacteria are known to be able to thrive successfully under extreme environmental constraints due to their mucilage production, motility or the production of akinetes in the case of some heterocyst-differentiating cyanobacteria (Murik et al., 2017; Ručová et al., 2018).

The dominance of coccoid, *Acaryochloris*-like cyanobacteria in the endolithic sample and its absence in soil samples seems to indicate a high level of specialization and adaptation to this type of environment. Indeed, as stated by Partensky et al. (2018) chl *d* allows *Acaryochloris* to thrive in (micro)habitats enhanced by light radiations other than the visible spectrum, remarkably those from the infrared region. The isolation of any strain would be of great relevance to help shed some light about the possible presence of this pigment (and in its eventual ecophysiological role) in the detected *Acaryochloris*-like cyanobacterium, which seems to be an important component of polar, endolithic communities. The existence of aridity-associated phylotypes, as suggested by the data presented, was already documented for other cold-deserts (Pointing et al., 2007). Inversely, actinobacteria abundance increases from about 20% in wet soils to roughly 50% in the driest samples and also with higher pH (Figure 3A and Table 1). This pattern suggests that actinobacteria is favored with the decrease in moisture content, contrary to cyanobacteria. A similar shift was observed by Takebayashi et al. (2007) from members of Proteobacteria to actinobacteria with decreasing in water availability. Germination and growth at 0.5 Aw has been previously reported for actinobacteria (Evans et al., 2014; Stevenson and Hallsworth, 2014) and Barnard et al. (2013) have shown that actinobacteria relative abundance increase with desiccation. Interestingly, and in contrast with what we observed, it has been shown that the relative abundance of actinobacteria of Namib desert increased with an increase in moisture (Armstrong et al., 2016) while the opposite has been shown to occur for Chihuahuan desert (Clark et al., 2009). Recent studies revealed the major predictors of moisture preferences are not phylogeny but physiological traits (Lennon et al., 2012), which can explain such contradictory observations. Also, soils with high pH usually support higher relative abundances of actinobacteria and lower of Acidobacteria when compared to more acidic soils (Fierer et al., 2012a). A comprehensive study with 88 different soils revealed a positive correlation between soil pH and actinobacteria abundance (Lauber et al., 2009), as we report in this study. The shift observed within actinobacteria distribution across the soil transect studied, might be a result not only of a decrease in water availability but a combination with pH increase.

Among Actinobacteria, families *Sporichthyaceae*, *Euzebyaceae*, *Patulibacteraceae*, and *Nocardioidaceae* were the most abundant (Figure 3C). The genus *Rubrobacter* (Pointing et al., 2009) was present in all samples, with a higher frequency in endolithic and in the last three samples of the transect. Previous studies have detected *Rubrobacter* in Dry Valleys soils (Aislabie et al., 2013; Wei et al., 2016), and the observed distribution suggests that this genus might be widely adapted to this environment.

Members of *Sporichthyaceae* family, with a higher distribution in sample T5, have been reported from Dry Valleys soil (Van Goethem et al., 2016) and cryptoendolithic communities (de la Torre et al., 2003) as well as Atacama desert soils (Idris et al., 2017). The distribution of *Patulibacteraceae* and *Nocardioidaceae* families increased in samples with lower water availability. Although at low frequencies, the *Nocardioides* genus was detected in all soil samples, suggesting, as for *Rubrobacter*, an important role for this genus in this arid environment. In fact, members of *Nocardioides* genus have been previously found in other desert soils, as Atacama (Idris et al., 2017) and Badain Jaran (Sun et al., 2018) deserts, which have inclusively led to novel species being isolated (Tuo et al., 2015).

Members of *Euzebyaceae* were detected particularly in END and T3 sample (Figure 3C), which is corroborated by previous works that have found the presence of members of the *Euzebya* genus in Dry Valleys hypolithic and endolithic communities (Van Goethem et al., 2016). In Atacama desert, their presence was also detected in endolithic microhabitats (Meslier et al., 2018). According to the literature, *Euzebya* seems to be highly adapted to endolithic and hypolithic environments, however, our study seems to be the first that detected the presence of this genus in the Dry Valleys soils. Remarkably, the endolithic sample was the only among those studied to harbor actinobacteria affiliated with *Streptomycetaceae*, a family commonly found and retrieved by cultivation from Dry Valleys soils samples (Cameron et al., 1972; Babalola et al., 2009).

### Culture-Dependent Isolation and Diversity of Actinobacterial Strains

Culture-based studies on Dry Valleys have initially proposed a dominance of a small number of aerobic groups, and few anaerobic isolates for endolithic (Friedmann, 1982) and edaphic habitats. Although, nowadays Antarctic soils have been extensively studied by culture-based approaches, these studies have still retrieved only a small number of bacterial phyla, from which only a small fraction of genera have been cultivated (Lambrechts et al., 2019).

However, molecular-based phylogenetic studies have revealed microbial diversity of Antarctic Dry Valley as remarkably high (Smith et al., 2006). At least 14 different bacterial phyla have been described from Dry Valleys bacterial lithic communities – dominated by Acidobacteria, Actinobacteria and Bacteroidetes (Cary et al., 2010). Still, culture-based studies have in general, retrieved some specific genera of the actinobacteria phylum such as *Arthrobacter*, *Brevibacterium*, *Corynebacterium*, *Micrococcus*, *Nocardia*, and *Streptomyces* (Johnson et al., 1972). Due to the low cultivable fraction of the microbial richness [typically below 1% (Epstein, 2013)], different strategies to improve the culturability of microorganisms have started to be used, including pre-treatment strategies and oligotrophic media (Xiong et al., 2013), which have provided fruitful results, in particular in Antarctic ecosystems (van Dorst et al., 2016; Pulschen et al., 2017; Tahon and Willems, 2017; Tahon et al., 2018). In fact, previous studies have revealed Antarctic edaphic bacteria resistant to cultivation but recently, Pulschen et al. (2017) has shown that it is possible to grow recalcitrant bacteria from Antarctic soils by using longer incubation periods, lower temperatures and oligotrophic media.

In the present study, different culture isolation strategies, including mimicking of oligotrophic conditions and application of selective pre-treatments were used, in order to isolate actinobacteria strains from Victoria Valley samples. Attempts to isolate actinobacteria were only successful in sample T5, where we used two different pre-treatments – heat shock and incubation with antibiotics. Eleven actinobacterial strains were isolated and identified in this study (Table 2), and they all belonged to the Micrococcales order, affiliated with two different families (*Micrococcaceae* and *Dermacoccaceae*) and four different genera (*Micrococcus*, *Kocuria*, *Dermacoccus*, and *Flexivirga*).

**Table 2:** Summary of obtained isolates.

Isolate	Accession number	Closest relative (identified species)	Number of obtained isolates	Accession number (NCBI)	Query cover (%)	Identity (%)	Isolation source	Phylum
*Micrococcus* sp. strain AT1	MH741266	*Micrococcus yunnanensis* strain YIM 65004	1	NR_116578.1	100	99	Soil–T5^1^	Actinobacteria
*Micrococcus* sp. strain AT2	MH741267	*Micrococcus yunnanensis* strain YIM 65004	1	NR_116578.1	100	99	Soil–T5^1^	Actinobacteria
*Dermacoccus nishinomiyaensis* strain AT4	MH741268	*Dermacoccus nishinomiyaensis* strain JPR-06	1	HE716946.1	100	99	Soil–T5^1^	Actinobacteria
*Dermacoccus nishinomiyaensis* strain AT6	MH741269	*Dermacoccus nishinomiyaensis* strain A-153	1	MF952731.1	100	99	Soil–T5^1^	Actinobacteria
*Kocuria* sp. AT6(2)	MH741270	*Kocuria rhizophila* strain TA68		NR_026452.1	100	99	Soil–T5^1^	Actinobacteria
*Micrococcus* sp. strain AT7	MH741271	*Micrococcus yunnanensis* strain YIM 65004	1	NR_116578.1	100	99	Soil–T5^1^	Actinobacteria
*Micrococcus* sp. strain AT9	MH741272	*Micrococcus aloeverae* strain AE-6	1	NR_134088.1	100	99	Soil–T5^1^	Actinobacteria
*Kocuria rhizophila* strain AT14	MH741273	*Kocuria rhizophila* strain TA68 16S	1	NR_026452.1	100	99	Soil–T5^2^	Actinobacteria
Micrococcus sp. Strain AT19	MH741274	*Micrococcus yunnanensis* strain YIM 65004	2	NR_116578.1	100	99	Soil–T5^3^	Actinobacteria
*Flexivirga* sp. Strain AT20	MH741275	*Flexivirga endophytica* strain YIM 7505	1	NR_151942.1	100	98	Soil–T5^3^	Actinobacteria
*Kocuria* sp. strain AT22	MH741276	*Kocuria rhizophila* strain TA68	1	NR_026452.1	100	99	Soil–T5^2^	Actinobacteria
*Leptolyngbya frigida* strain AR4-GA-1C	MH742927	*Leptolyngbya frigida* ANT.L52B.3	1	AY493612.1	100	99	Soil – T6^4^	Cyanobacteria
*Leptolyngbya frigida* strain AR3-EB-1A	MH742928	*Leptolyngbya frigida* ANT.L52B.3	2	AY493612.1	100	99	Soil – T1^4^	Cyanobacteria
*Nodosilinea* sp. strain TM-3.1	MH742933	*Leptolyngbya antarctica* ANT.LAC.1	4	AY493588.1	100	98	Soil – T3^4^	Cyanobacteria
*Nodosilinea* sp. strain TM-3.5	MH742934	*Phormidesmis priestleyi* ANT.LACV5.1	1	AY493581.1	100	98	Soil – T1^4^ and T3^4^	Cyanobacteria
*Leptolyngbya frigida* strain AR3-B-1A	MH742929	*Leptolyngbya frigida* ANT.L52B.3	1	AY493612.1	98	98	Soil – T1	Cyanobacteria
Unidentified Synechococcales strain AR3-H-2A	MH742931	*Phormidesmis priestleyi* ANT.LACV5.1	1	AY493586.1	100	98	Soil – T1^4^	Cyanobacteria
*Plectolyngbya hodgsonii* strain AR4-AB-1B	MH742930	*Plectolyngbya hodgsonii* ANT.LG2.1	1	AY493615.1	100	99	Soil – T6^4^	Cyanobacteria
*Leptolyngbya frigida* strain AR3-EA3	MH742932	*Leptolyngbya frigida* ANT.L52B.3	1	AY493612.1	100	100	Soil – T1^4^	Cyanobacteria
*Nodosilinea* sp. LEGE 13457	KT951670.1	*Leptolyngbya antarctica* ANT.LAC.1	1	AY493588.1	100	99	END^4^	Cyanobacteria
*Nodosilinea* sp. LEGE 13458	KU951755.1	*Leptolyngbya antarctica* ANT.LAC.1	1	AY493588.1	100	99	END^4^	Cyanobacteria

*The isolates non-identified, clonal strains and strains in isolation process were excluded from the Table. ^1^Isolation in AIA medium, at 28°C, PT1, dilution 10^−1^. ^2^Isolation in AIA medium, at 28°C, PT2, not diluted. ^3^Isolation in SCN medium, at 28°C, PT2, substrate inoculated. ^4^Isolation in Z8 medium, at 19°C.*

**FIGURE 5:**
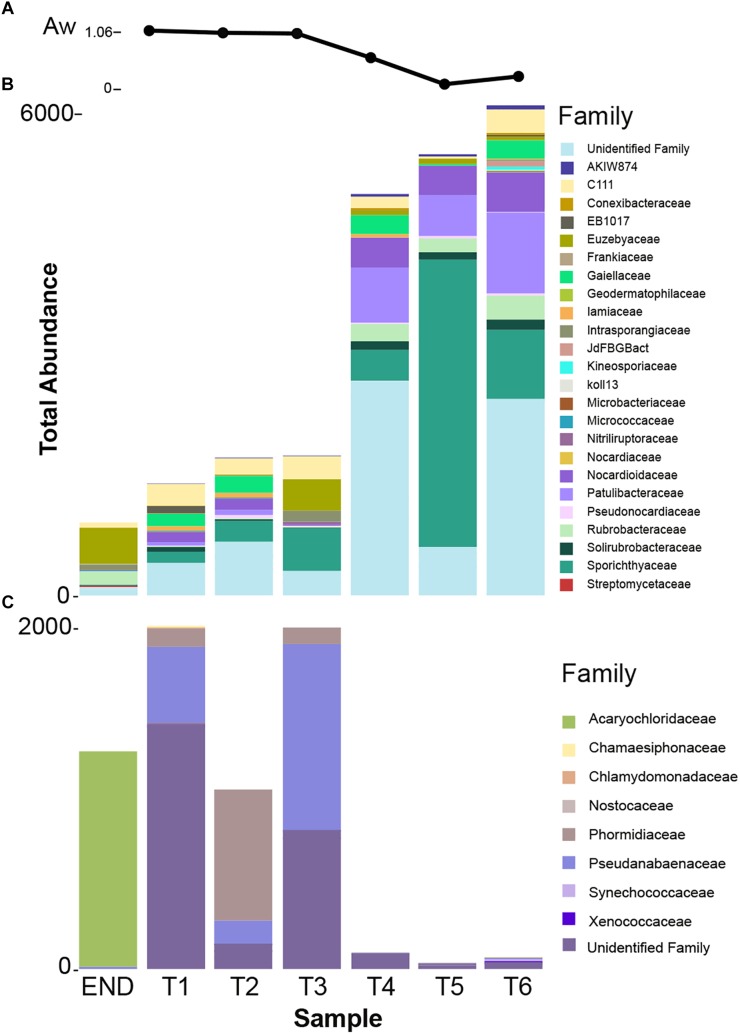
**(A)** Water availability (Aw) distribution chart per sampling point. **(B)** Taxonomy summary bar chart of total frequency of actinobacterial families present in each sampling point. **(C)** Taxonomy summary bar chart of total frequency of cyanobacterial families present in each sampling point.

**FIGURE 6:**
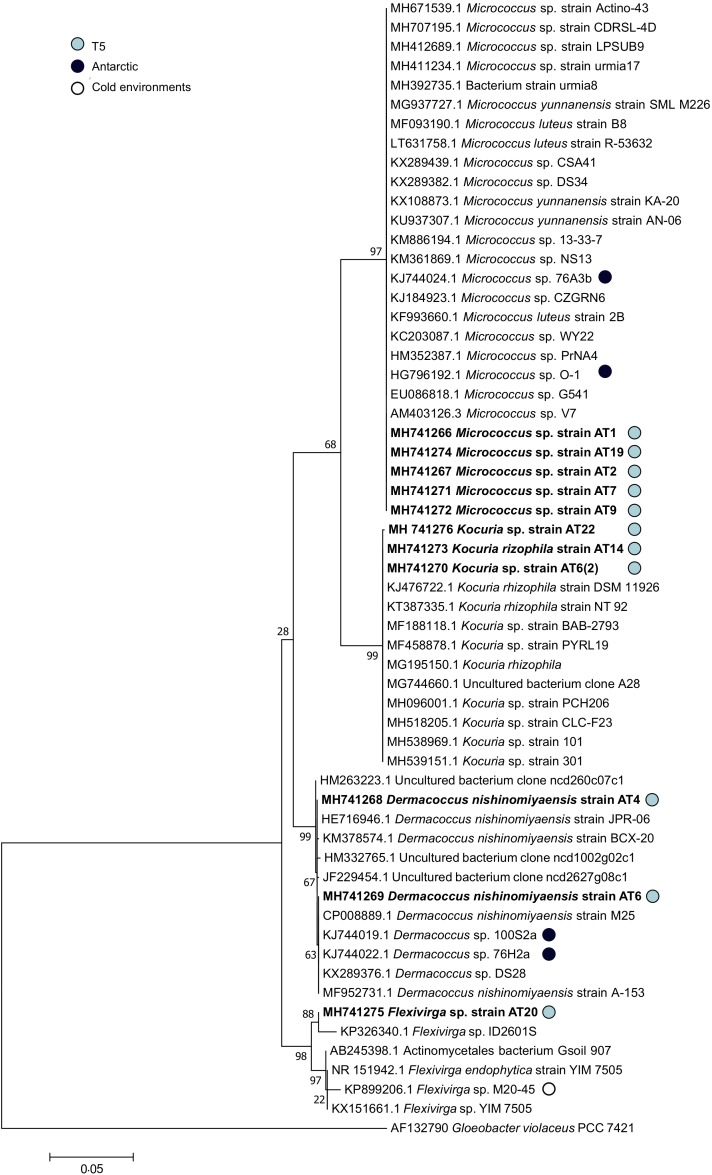
Phylogenetic tree of the 16S rRNA gene nucleotide sequences of the obtained actinobacterial isolates and their closest matches at NCBI 16S database. The evolutionary history was inferred by using the Maximum Likelihood method based on the Tamura–Nei model. A discrete Gamma distribution was used to model evolutionary rate differences among sites [5 categories (+*G*, parameter = 0.4569)]. The tree is drawn to scale, with branch lengths measured in the number of substitutions per site. The analysis involved 59 nucleotide sequences. Sequences of strains isolated in Antarctic or cold-environments are highlighted and the sequences obtained in this study are in bold.

According to pyrosequencing data, the dominant actinobacteria family in sample T5 was *Sporichthyaceae*, followed by *Patulibacteraceae*, *Nocardioidaceae*, and *Rubrobacteraceae* (Figure 3C). For the *Sporichthyaceae* family, there are no records of cultivation isolates in Antarctica. Curiously, accordingly to the pyrosequencing data, *Micrococcaceae* were not found in sample 5 and *Dermacoccaceae* were not detected in any sample (Figure 5B). Bacterial species from *Micrococcaceae* family are commonly retrieved from Antarctic culture-based studies (Johnson et al., 1972; Liu et al., 2000; Cary et al., 2010). The obtained isolates were assigned to two different and less common genera from this family – *Micrococcus* and *Kocuria*. The *Kocuria* genus has resulted from the phylogenetic and chemotaxonomic division of *Micrococcus* genus and both include species isolated in Antarctica (Liu et al., 2000; Reddy et al., 2003). Despite being considered a less common genus, members of *Kocuria* genus are recurrently isolated across desert soils (Gommeaux et al., 2010; Schulze-Makuch et al., 2018), inclusive new species (Li et al., 2006). Species from the *Dermacoccus* genus have also been previously isolated from Galindez Island, maritime Antarctica (Vasileva-Tonkova et al., 2014). One of the reasons behind *Micrococcaceae* cultivation amenability can be related to the production of cyst-like resting forms. It has been shown that *Micrococcus* species from permafrost harbor such cyst-like cells, that provide protection to adverse external factors and are responsible for their survival under prolonged exposure to subzero temperatures (Soina et al., 2004). In addition, another study revealed that members of *Micrococcaceae* family have higher growth rates in water addition samples, when comparing to other bacterial groups (Schwartz et al., 2014), suggesting that members of this family are adapted to intend for transient water inputs in arid soils. For the remaining genus – *Flexivirga* – there is no report for previous isolation in Antarctica, however, one of the 5 closest hits at NCBI (*Flexivirga* sp. M20-45) was isolated from an alpine forest soil by Franca, L. and Margesin, R. (unpublished). In the phylogenetic tree (Figure 6) the isolate AT20 groups with the strain *Flexivirga* sp. ID2601S, retrieved from an evaporation core by Kim et al. (unpublished). According to the phylogenetic analysis and the 16S rRNA similarity, the isolate AT20 might represent a new species from the *Flexivirga* genus.

### Culture-Dependent Isolation and Diversity of Cyanobacterial Strains

Cyanobacteria dominance and adaptive success in Antarctica is well known (Vincent, 2007; Quesada and Vincent, 2012). They thrive particularly in lakes and ponds through the establishment of benthic microbial mats (Vincent, 2000). Pioneer studies on Dry Valleys cyanobacterial distribution, have proven that the majority of cryptoendolithic cyanobacteria-dominated communities belong to the *Phormidium* genus (Friedmann, 1986).

Here, attempts were made to isolate cyanobacteria from soil and endolithic rock samples. In total 10 cyanobacterial strains from the order Synechococcales were obtained (Table 2), distributed by 3 different genera (*Nodosilinea*, *Leptolyngbya*, *Pectolyngbya*, and an unidentified Synechococcales).

**FIGURE 7:**
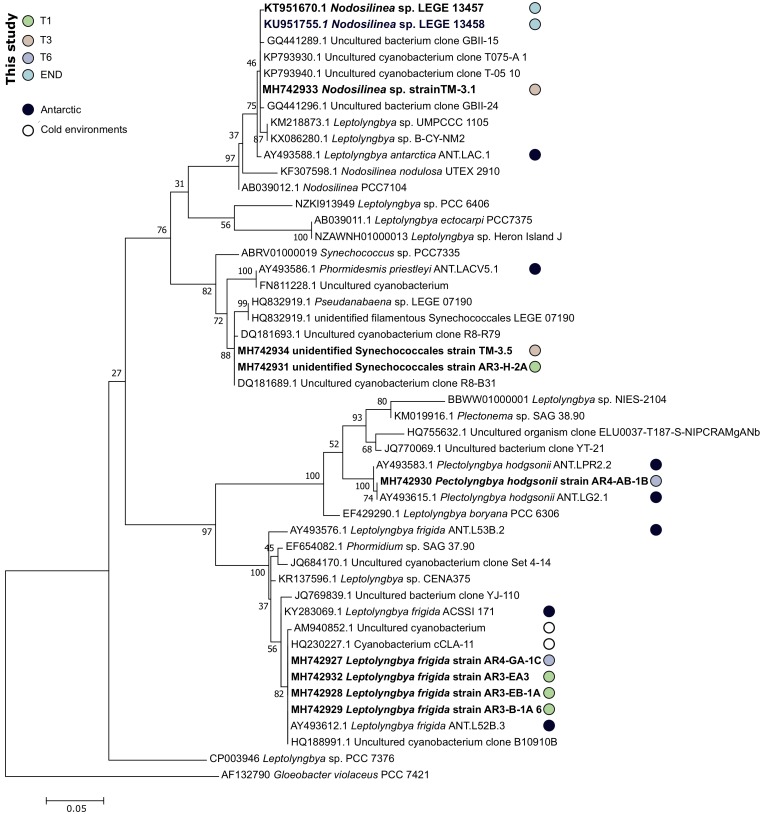
Phylogenetic tree of the 16S rRNA gene nucleotide sequences of the obtained cyanobacterial isolates and their closest matches at NCBI 16S database. The evolutionary history was inferred by using the Maximum Likelihood method based on the General Time Reversible model. A discrete Gamma distribution was used to model evolutionary rate differences among sites [5 categories (+*G*, parameter = 0.2861)]. The rate variation model allowed for some sites to be evolutionarily invariable ([+*I*], 34.68% sites). The tree is drawn to scale, with branch lengths measured in the number of substitutions per site. The analysis involved 48 nucleotide sequences. Sequences of strains isolated in Antarctic or cold-environments are highlighted and the sequences obtained in this study are in bold.

From END sample, it was only possible to isolate two clonal strains (*Nodosilinea* sp. LEGE 13457 and LEGE 13458) with high phylogenetic (Figure 7) and morphological (Supplementary Figure S2) similarities to one *Leptolyngbya antarctica* strain previously isolated from a benthic microbial mat in Dry Valleys and eastern Antarctic lakes (Taton et al., 2006). Although they were identified with over 99% similarity to *L. antarctica* ANT.LAC.1, the phylogenetic analysis indicates that LEGE 13457 and LEGE 13458 strains fit within the clade harboring *Nodosilinea* strains and is placed away from the reference strain *Leptolyngbya boryana* PCC6306 (Figure 7). This apparent inconsistency is due to the current status of the taxonomy of cyanobacteria, which is in a protracted process of revision (Komárek, 2016; Walter et al., 2017). Thus, our findings suggest that a taxonomic revision of the species *L. antarctica* (West and G.S.West) Anagnostidis and Komárek (1988) is needed, something that was already demonstrated by Taton et al. (2006). According to the pyrosequencing data, over 40% of the total bacterial abundance of the END sample was composed of strains from *Acaryochloris* genus and only 0.4% of *Leptolyngbya*, however, and despite our efforts we were not able to properly isolate any strain from *Acaryochloris* genus. As abovementioned, this would be very important to better explore the role of chl *d* for the successful adaptation of this cyanobacterium in endolithic habitats from Antarctica, in a similar manner as it was exposed for marine counterparts (Chan et al., 2007). Also, sequences from *Nodosilinea* strains were not detected by the pyrosequencing technology. Interestingly, *L. antarctica* ANT.LAC.1 is the only strain from Antarctica present on the clade harboring the referred isolated strains (LEGE 13457 and LEGE 13458; Figure 7). *L. antarctica* has been detected from several environments not exclusively in Antarctica, and has been found as a dominant OTU in Arctic soil crusts (Pushkareva et al., 2015).

Attempts were made to isolate cyanobacteria from three soil transect samples, T1 – the sample with highest water availability, T3 and also T6 – with an arid soil-type habitat. From sample T1, three strains identified as *Leptolyngbya frigida* and one unidentified Synechococcales were obtained. According to the phylogenetic tree, the unidentified Synechococcales strain is most closely related to uncultured cyanobacterium strains from Antarctica, and according to the matrix of distances it only shares 97.5% of similarity to *Phormidesmis priestleyi* ANT.LACV5.1, suggesting it can represent a new species. In agreement with the cultivation results, the pyrosequencing data revealed that the dominant genera corresponded to *Leptolyngbya, Pseudanabaena*, and *Phormidium*, however, *Phormidesmis* genus remained undetected (Figure 4). Interestingly, from sample T3, a identical strain (100% similarity at the 16S rRNA) from the one obtained in sample T1, unidentified Synechococcales strain AR3-H-2A and a *Nodosilinea* sp. strain, with high identity to the ones isolated in END sample, were retrieved. According to the pyrosequencing data, the sample T3 contained well-represented strains from *Pseudanabaenaceae* family (Figure 3B) and a lower distribution of the *Phormidium* genus (Figure 4). Concerning the sample with lowest water availability – sample T6 – two strains identified as *Plectolyngbya hodgsonii* and *L. frigida* were obtained. From the phylogenetic tree (Figure 7) is possible to verify that the *obtained Pectolyngbya* isolates group together in a subclade formed only by Antarctic strains. The pyrosequencing data revealed that this sample only contained 0.1% of cyanobacteria from the *Synechococcaceae* family and did not detect any from *Leptolyngbyaceae* to which the isolated strains were affiliated (Figure 5C).

All the 16S rRNA gene sequences from obtained strains are more closely-related to other Antarctic or cold-environment cyanobacteria strains (Figure 7). Particularly, the clade of *L. frigida* is composed in its majority of strains isolated in Antarctica or cold-environments. Notably, most of the closest relatives at NCBI correspond all to the same study (Taton et al., 2006) that assessed the cyanobacteria diversity in Antarctic lakes, including in the Dry Valleys.

### Culture-Dependent vs. Culture-Independent Approach

Over 99% of microorganisms from the environment are recalcitrant to cultivation in the laboratory (Kaeberlein et al., 2002) and the revolution of HTS techniques has opened an array of opportunities with new discoveries and access to previously unknown and uncultivable diversity. However, a few bottlenecks such as limited detection of minority populations (Lagier et al., 2012), difficulty to discriminate the lowest taxonomic level and the fraction of unassigned sequences (Rinke et al., 2013) have renewed the interest in bacterial cultivation practices for “non-cultivable” species. Together with the development of new approaches to retrieve bacteria previously considered as uncultivable, the rebirth of culture in microbiology (Kaeberlein et al., 2002; Lagier et al., 2018) has emerged. The use of culture media and diffusion chambers to simulate natural environments (Kaeberlein et al., 2002), co-culture (Stewart, 2012) and most recently culturomics (Lagier et al., 2016) have yielded great improvements in culturability. Actually, the combination of HTS methods with culture-dependent techniques has started to be used to identify new bacterial species (Ma et al., 2014).

Here, a combined approach including HTS and culture-dependent techniques (including mimetization of the natural conditions and use of pre-treatments) was employed. In this study, the isolation of the same cyanobacterial species (including clonal strains) from different samples and different microenvironments, suggests, as already reported (Taton et al., 2006), that the cultivation conditions may have selected for some specific genera. While, OTU-level rarefaction curve of END sample has reached a plateau (Supplementary Figure S1), indicating that we have captured most of the bacteria diversity, 50% of the isolated strains were not detected by HTS sequencing technology. Factors such as bias in DNA extraction (Zielińska et al., 2017) due to the protocol used, differential PCR amplification or the different distribution of rRNA operons in the different bacteria can influence the proportion of rRNA phylotypes (Klappenbach et al., 2000).

Concerning the isolation of actinobacteria, the combination of pre-treatments with culture conditions clearly dictated the success in isolation of T5 sample. Interestingly, none of the isolated strains from this study that were affiliated with *Micrococcaceae* and *Dermacoccaceae* were represented in the pyrosequencing data set of samples from which isolates were retrieved (T5, Figure 3C). As already demonstrated by Pulschen et al. (2017) and Tahon and Willems (2017), it is possible to retrieve recalcitrant bacteria from Antarctic samples by adopting some simple approaches such as longer incubation periods, use of low-temperatures and oligotrophic media. By applying these approaches Tahon and Willems (2017) were able to obtain at least 12 representatives of novel genera or families and two potential first cultured isolates of novel taxa.

It is important to note that due to samples being preserved at −80°C on life-guard solution, some diversity may have not been recovered due to a decrease in viability associated with storage in this solution and temperatures. In addition, other variables can influence the ability of bacteria to grow, from culture media composition (Xiong et al., 2013) to more complex requirements, as the presence of specific growth signals (Lewis et al., 2010) or dependency on other microorganism(s) (Davis et al., 2011). Also, the percentage of active members of the community that can be cultivable is usually low. It has been previously suggested that in arid soils, such as Antarctica Dry Valleys, the cyanobacterial populations are not actively growing (Aislabie et al., 2013), as they originate probably from wind dispersion (Michaud et al., 2012).

Previous studies have also demonstrated that the complementarity of culture-dependent and independent approaches is represented by only a fraction of species detected concomitantly (Lagier et al., 2012; Pudasaini et al., 2017; Tahon and Willems, 2017). The low complementarity can be explained by the limitations presented of both approaches. Further Carini et al. (2017) have shown that relic DNA accounts for about 40% of the prokaryotic 16S rRNA amplified genes. In colder soils, DNA from non-viable cells can persist even for longer periods, thus an inflation in bacterial diversity might be one of the reasons for the reduced overlap observed.

As already suggested, a combined approach using HTS to guide the culture-based isolation process (Babalola et al., 2009) and the use of pre-treatments and specific culture media (Pulschen et al., 2017), can improve the identification and culture retrieval of new bacterial species.

The limited sampling sites covered by this study can result in some bias. Although might reduce the confidence of our hypothesis, most of the results presented are supported by previous studies that should, however, be further explored in future sampling campaigns in the Dry Valleys.

## Conclusion

This study combined pyrosequencing and cultivation techniques to assess the actinobacteria and cyanobacteria diversity of Dry Valleys microenvironments. The effect of environmental parameters on the distribution of these communities, in particular along a soil transect with a gradient of water availability, was also evaluated. This study highlights the capacity of Dry Valleys prokaryotic communities to thrive below thresholds that are considered to be life-limiting. The major role of actinobacteria and cyanobacteria, the dominant heterotrophs and phototrophs, in Dry Valleys ecosystem is supported by their distribution across environmental gradients.

Our findings are in agreement with other studies, by demonstrating that Dry Valleys bacterial diversity and abundance is driven by environmental forces. The effect of one particular environmental parameter – water availability – was evaluated and a clear shift between microbial communities was registered. This shift was characterized by a pattern of phyla replacement as distance to the water source increased, likely resulting from a shift in habitat from high moisture soils to open arid soils.

Our results revealed that the type of habitat (endolithic vs. soil) dramatically constrains the bacterial community composition, characterized by a clearly distinct taxonomic and phylogenetic composition between both characterized habitats. Cyanobacteria dominated over the remaining phyla in the endolithic environment. The gradient of water availability (Figure 5A) and pH seems (Table 1) to dictate the distribution of cyanobacteria and actinobacteria, suggesting that actinobacteria is favored with the decrease in moisture content and increase in pH, contrary to cyanobacteria. However, caution is necessary when extrapolating from these results since a reduced number of samples were analyzed here. Finally, our study further illustrates the importance of combining cultivation and sequencing techniques. Indeed, despite the power of HTS technologies, we show that culture-dependent methods employed in this study were able to retrieve taxa that were not detected in any of the pyrosequencing data. By combining the two approaches, we have improved the coverage of the diversity present in the samples and were able to retrieve both abundant and rare members of the communities. The isolation of microorganisms from this environment remains challenging, and future work will include further optimization of isolation strategies and culture conditions.

## Author Contributions

AR, MC, PL, and CM designed and conceived the research study and experiments. AR, FR, TM, HR, MB, and JS developed the experimental work. AR, AS, VR, and PL analyzed the data. AR wrote the main manuscript text with support of CM, MC, VR, PL, and MB. SC and CL coordinated Antarctica sampling campaign. All authors improved, reviewed, and approved the final manuscript.

## Conflict of Interest Statement

The authors declare that the research was conducted in the absence of any commercial or financial relationships that could be construed as a potential conflict of interest.
