# Supplementary material for: Actinobacteria and Cyanobacteria Diversity in Terrestrial Antarctic Microenvironments Evaluated by Culture-Dependent and Independent Methods
Source: Front Microbiol. 2019 May 31;10:1018. doi: 10.3389/fmicb.2019.01018 (PMC6555387; doi:10.3389/fmicb.2019.01018)
Supplement: Supplementary file 1 [file Data_Sheet_1.pdf]

Supplementary Material

Actinobacteria and Cyanobacteria Diversity in Terrestrial Antarctic Microenvironments Evaluated by Culture-Dependent and Independent Methods

Adriana Rego, Francisco Raio, Teresa P. Martins, Hugo Ribeiro, António G.G. Sousa, Joana Séneca, Mafalda S. Baptista, Charles K. Lee, Craig S. Cary, Vitor Ramos, Maria F. Carvalho, Pedro N. Leão and Catarina Magalhães*

*** Correspondence:** Catarina Magalhães: catarinamagalhaes1972@gmail.com

Supplementary Figures

Figure S1 – Alpha-diversity metrics computed using QIIME: observed OTUS, Chao1 and Phylogenetic diversity.

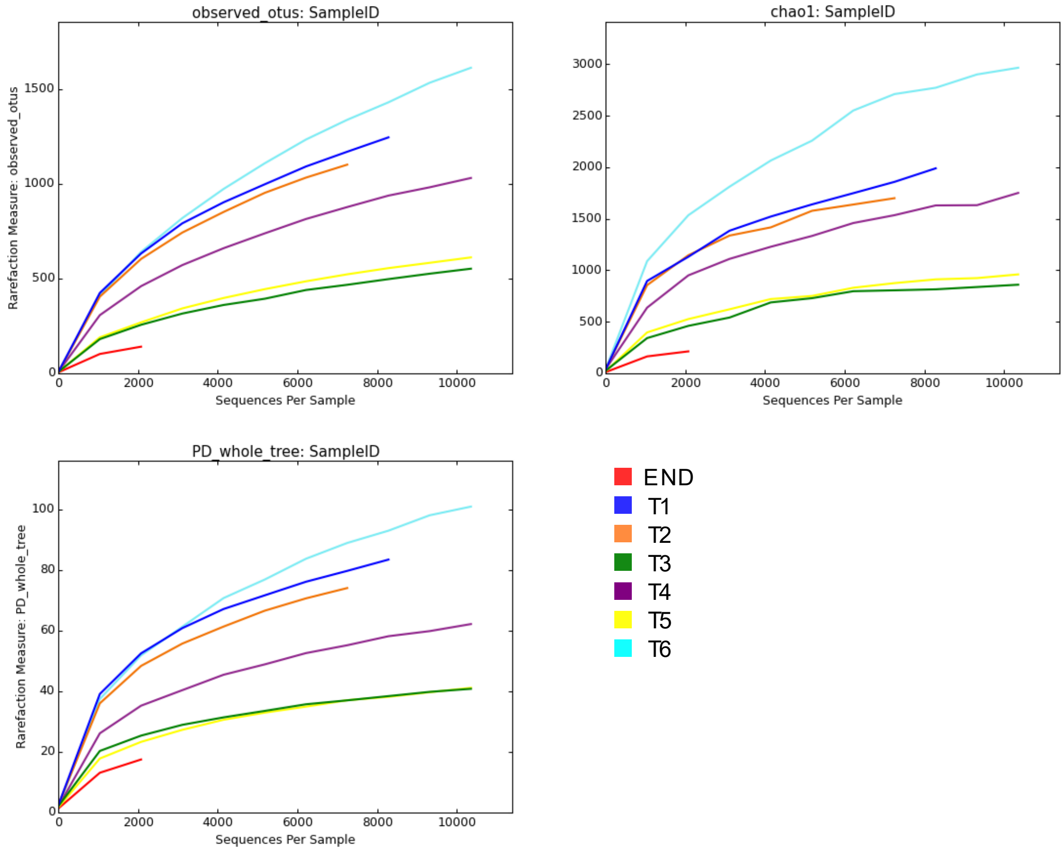

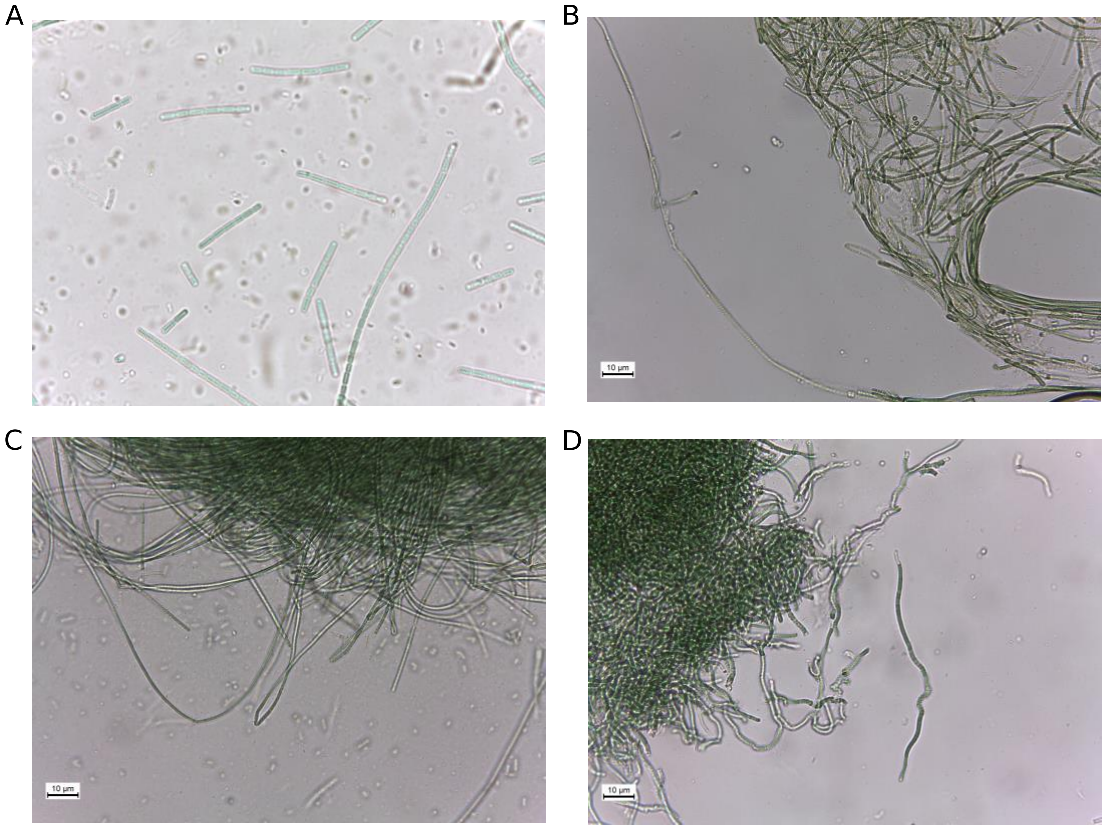

Figure S2 - Microphotographs obtained from a light microscopic (Leica DMLB) with the software Leica LAS EZ, from Cyanobacteria strains representatives of the diversity retrieved in this study. A- Microphotograph of *Nodosilinea* sp. strain TM-31 (representative of Nodosilinea sp. LEGE 13457 and 13458), at 1000x amplification, B – microphotograph of unidentified Synechococcales strain AR3H-2A at 400x amplification, C – microphotograph of *Leptolyngbya frigida* strain AR4-GA-1C, at 400x amplification, D – microphotograph of *Plectolyngbya hodgsonii* strain AR4-AB-1B, at 400x amplification.

Supplementary Tables

Table S1 – Number of sequences of the samples in study.

	**Number of sequences**
Initial (all samples)	180499
After quality filtering (all samples)	71447

Sample T1	9273
Sample T2	7692
Sample T3	11893
Sample T4	11712
Sample T5	10357
Sample T6	17570
Sample END	2959
